# Prehospital Detection of Life-Threatening Intracranial Pathology: An Unmet Need for Severe TBI in Austere, Rural, and Remote Areas

**DOI:** 10.3389/fneur.2020.599268

**Published:** 2020-10-30

**Authors:** Mark D. Whiting, Bradley A. Dengler, Carissa L. Rodriguez, David Blodgett, Adam B. Cohen, Adolph J. Januszkiewicz, Todd E. Rasmussen, David L. Brody

**Affiliations:** ^1^The Center for Neuroscience and Regenerative Medicine, Uniformed Services University of the Health Sciences and National Institutes of Health, Bethesda, MD, United States; ^2^Stephens Family Clinical Research Institute, Carle Foundation Hospital, Urbana, IL, United States; ^3^Department of Neurosurgery, Walter Reed National Military Medical Center, Bethesda, MD, United States; ^4^Johns Hopkins University Applied Physics Laboratory, Laurel, MD, United States; ^5^Department of Neurology, Johns Hopkins School of Medicine, Baltimore, MD, United States; ^6^Knowesis Inc., Gunpowder, MD, United States; ^7^Department of Surgery, Uniformed Services University of the Health Sciences, Bethesda, MD, United States; ^8^Department of Neurology, Uniformed Services University of the Health Sciences, Bethesda, MD, United States; ^9^Laboratory of Functional and Molecular Imaging, National Institute of Neurological Disorders and Stroke, Bethesda, MD, United States

**Keywords:** traumatic brain injury, ultrasound, near infrared spectroscopy, prehospital care, rural medicine

## Abstract

Severe traumatic brain injury (TBI) is a leading cause of death and disability worldwide, especially in low- and middle-income countries, and in austere, rural, and remote settings. The purpose of this Perspective is to challenge the notion that accurate and actionable diagnosis of the most severe brain injuries should be limited to physicians and other highly-trained specialists located at hospitals. Further, we aim to demonstrate that the great opportunity to improve severe TBI care is in the prehospital setting. Here, we discuss potential applications of prehospital diagnostics, including ultrasound and near-infrared spectroscopy (NIRS) for detection of life-threatening subdural and epidural hemorrhage, as well as monitoring of cerebral hemodynamics following severe TBI. Ultrasound-based methods for assessment of cerebrovascular hemodynamics, vasospasm, and intracranial pressure have substantial promise, but have been mainly used in hospital settings; substantial development will be required for prehospital optimization. Compared to ultrasound, NIRS is better suited to assess certain aspects of intracranial pathology and has a smaller form factor. Thus, NIRS is potentially closer to becoming a reliable method for non-invasive intracranial assessment and cerebral monitoring in the prehospital setting. While one current continuous wave NIRS-based device has been FDA-approved for detection of subdural and epidural hemorrhage, NIRS methods using frequency domain technology have greater potential to improve diagnosis and monitoring in the prehospital setting. In addition to better technology, advances in large animal models, provider training, and implementation science represent opportunities to accelerate progress in prehospital care for severe TBI in austere, rural, and remote areas.

## Introduction

Severe traumatic brain injury (TBI) is a leading cause of death and disability worldwide (1). In low- and middle-income countries, the incidence of severe TBI has risen with the use of motorized transport, while shifting demographics have resulted in more fall-related injuries in the aging population (2). In military personnel, exposure to blast is associated with unique pathological changes frequently accompanied by blunt force or penetrating trauma (3, 4). In the event of armed conflict with an advanced peer or near-peer adversary, severe injuries of all types are expected to be more common than in recent wars and casualty care complicated by delayed medical evacuation (5). Although severe injuries represent a small percentage of all TBI, they are associated with the highest levels of morbidity and mortality. Among survivors, there is a reduced life expectancy compared to the general population (6).

Severe TBI occurring in austere, rural, or remote settings presents special challenges above and beyond those of the injury itself. Those living in rural areas have a higher annualized incidence of TBI, and both total and prehospital mortality are higher when the injury occurs in a rural setting (7, 8). The fact that many rural hospitals in the U.S. are downsizing or closing exacerbates this problem. Nearly 47 million American's live more than 1 h away from trauma care (9), which is of particular relevance to severe TBI for which rapid diagnostics and treatments are required. Level I trauma facilities are even more dispersed, and sub-specialists such as neurosurgeons are concentrated in urban areas. Areas without ready access to high acuity intensive care have been referred to as “trauma deserts” (10). In the military, expectations that future operational environments will require far-forward troops to delay medical evacuation and treat injured personnel onsite for extended periods has led to the concept of prolonged field care (11).

The purpose of this Perspective is to challenge the notion that accurate and actionable diagnosis of severe brain injuries should be limited to physicians and other highly-trained specialists located at hospitals. In order to meet the needs of citizens living in rural areas throughout the world, as well as military personnel deployed in remote and resource-limited locations, the capability to recognize life-threatening intracranial pathologies after TBI must be placed firmly into the hands of prehospital providers. Advanced technologies and unconventional approaches are needed to manage patients for *hours* in rural environments and possibly *days* in wilderness medicine or military prolonged field care settings.

## Unmet Needs

### Diagnosis of Intracranial Bleeding and Other Intracranial Pathologies

Intracranial extra-axial bleeding, including subdural (SDH) and epidural hemorrhage (EDH), is a risk factor for increased mortality following severe TBI (12, 13). When diagnosis and treatment are delayed, mortality may be as high as 90%, the worst of any subgroup of patients with severe TBI (14–16). Additionally, the clinical course of patients with delayed traumatic hematomas is often unpredictable, with some deteriorating rapidly and others exhibiting no clinical change (17). In a hospital setting, detection of intracranial bleeding approaches 100% accuracy with CT. Outside of hospital settings equipped with CT and MRI, there are no approaches to reliably detect or assess these pathologies, and accurate detection of intracranial bleeding remains a critical barrier to reducing mortality in the absence of conventional neuroimaging. Thus, there is a great need for the ability to detect life-threatening intracranial bleeding in the prehospital setting. Moreover, monitoring intracranial pressure (ICP), cerebral autoregulation, and vasospasm may play a role in predicting outcome from severe TBI and can help drive interventions to stabilize patients awaiting transport to higher level facilities. Here we discuss potential applications of ultrasound and near-infrared spectroscopy (NIRS) for detecting life-threatening hemorrhage and monitoring cerebral hemodynamics (Table 1). Emphasis is placed on modalities that lend themselves to prehospital application in a remote or austere environment.

**Table 1:** Intracranial pathologies seen in severe TBI and potential diagnostic methods for the prehospital setting.

**Intracranial pathology**	**Potential diagnostic methods**	**Pro**	**Con**
Cerebral autoregulation	TCD-derived indices (18–23)	Non-invasive Portable	Operator dependent Non-continuous Not validated Limited by poor bone penetration Large learning curve Influenced by many different factors Time intensive
Traumatic vasospasm	TCD-derived pulsatility index (24–28)	Non-invasive Portable	Operator dependent Non-continuous Limited by poor bone penetration Large learning curve Influenced by many different factors Time Intensive
Intracranial pressure	Ultrasonography of ONSD (29–34)	Fast Non-invasive Portable Easy to acquire with minimal training (33)	Cannot be used with trauma to the globe Optimal cut-offs not established Lack of data on how long ICP is elevated before change in ONSD
	TCD—derived indices (35–40)	Non-invasive Portable	Operator dependent Significant training required with steep learning curve Limited by poor bone windows in some patients
Intracranial hematoma	Continuous wave NIRS (41–46)	Non-invasive Portable form factor[vanji] Currently FDA approved and marketed	Bilateral reference scan needed Sub-optimal specificity, especially in the setting of scalp hematoma
	Frequency domain NIRS	Non-invasive Single scan is sufficient (in contrast to CW NIRS, which needs bilateral reference scan)	Portable form factor does not currently exist Higher cost compared to CW NIRS

*TCD, transcranial Doppler; ONSD, optical nerve sheath diameter; NIRS, near infrared spectroscopy; CW, continuous wave; FD, frequency domain*.

#### Ultrasound

Ultrasound is a widely-used tool in emergency medicine and is a relatively low-cost and portable option for non-invasive imaging in a field setting. However, ultrasound has poor bone penetration and low resolution, thus limiting its application to acute intracranial insults. Nonetheless, developments in technology continue to broaden the spectrum of utility for transcranial ultrasound, including real-time evaluation of intracranial blood flow dynamics, detection of vasospasm, and monitoring of ICP (18, 19).

Cerebral autoregulation, the ability to regulate and maintain blood flow following changes in cerebral perfusion pressure (CPP), is frequently impaired following TBI (47). Identification of impaired autoregulation may help identify patients likely to benefit from targeted therapies such as aggressive blood pressure management. Cerebral autoregulation is typically measured via the pressure reactivity index, and some studies indicate that continuous monitoring during the acute phase of TBI may lead to improved prognosis (48, 49). Previous work has established that transcranial Doppler (TCD) ultrasound-derived indices can be used to non-invasively assess cerebral autoregulation (20, 21). Despite poor bone penetration, TCD exploits thinner skull regions to assess cerebral blood flow, and some studies have extended these methods to severe TBI. For instance, TCD measurement of blood flow velocity correlated with either CPP or arterial pressure has been used to establish indices that distinguish normal from impaired cerebral autoregulation. Using this method, Sorrentino et al. established critical values of TCD-derived autoregulation to demonstrate a relationship with mortality and poor outcome (22). Subsequently, Budohoski et al. used TCD to examine systolic, mean, and diastolic flow velocity in 300 patients with TBI and found that systolic indices demonstrated the strongest association with outcome (23).

Traumatic vasospasm is prevalent in civilian and military TBI and is predictive of worse neurological outcome (24, 50). Frequently seen in conjunction with SDH, traumatic subarachnoid hemorrhage, and contusion, vasospasm typically occurs within 2 days of injury and is a predictor of delayed cerebral ischemia (50). Some authors have proposed that serial monitoring may identify patients at the greatest risk for ischemic injury and thus intensive treatment (25). Lee et al. demonstrated that TCD alone could detect vasospasm that predicts worse outcome (26). Following wartime TBI, daily TCD was used to monitor cerebral hemodynamic changes including vasospasm and intracranial hypertension in military service members with combat-related injuries. Mild, moderate, and severe levels of vasospasm were detected in 37, 22, and 12% of patients (27). TCD has also been used to screen for intracranial hypertension and abnormal CPP. Specifically, the TCD-derived pulsatility index is thought to correlate with both ICP and CPP (28, 35, 36), although other reports question this method (37). In children with severe TBI, TCD predicts intracranial hypertension and abnormal CPP (38), although its utility for detecting changes in ICP may be limited to the first 24 h after injury (39). In adults with severe TBI, mean blood flow and pulsatility index of the middle cerebral artery were shown to correlate with ICP and CPP and predicted 6 months outcome (40).

In addition to early detection of altered cerebral hemodynamics, ICP can be inferred from ultrasonography of the optical nerve sheath diameter (ONSD), which increases as a direct result of increasing ICP (29, 30). In one study of 63 patients with subarachnoid or primary intracerebral hemorrhage, an ONSD of 5.2 mm was determined the optimum cutoff to predict an ICP > 20 mmHg with 93.1% sensitivity and 74% specificity; furthermore, a rapid change in the ONSD was observed following CSF drainage (31). Additionally, the multitude of studies have shown improved specificity and sensitivity as the ONSD increases above the cut-off values; a meta-analysis of six studies including 231 patients found cross study sensitivity of 90% and specificity of 85% for detection of raised ICP (32). The technique can be effectively taught in a 4-h training session, even to novice users (33). However, some studies have questioned the reliability of the ONSD method (34), and TCD-based approaches have shown greater diagnostic accuracy in some studies (51, 52).

While these studies demonstrate the potential utility of ultrasound in TBI applications, nearly all were conducted in hospital settings where ultrasound was used as an adjunct to traditional monitoring techniques. Rarely has ultrasound been used as a stand-alone diagnostic or to guide treatment, although a preliminary report suggests that ultrasound can be used in the prehospital setting to detect impaired cerebral perfusion (53), and a portable, ruggedized ultrasound system is currently being developed under a U.S. military contract (54). To date, there are no widely-accepted or validated criteria for ultrasound assessment of vasospasm, cerebral autoregulation, or ICP. At present, no ultrasound-based device is likely to be effective for detection of intracranial hemorrhage, and it has not yet been demonstrated that key hardware components for through-skull imaging can be made into a hand-held form factor suitable for field use. To deploy ultrasound successfully as a prehospital diagnostic for severe TBI, future prospective studies are needed to refine operational parameters, determine appropriate thresholds for intervention, and explore novel approaches that remain largely untested in TBI, including contrast-enhanced (55) and shear-wave ultrasound (56, 57), along with fusing ultrasound data with other sensor modalities and clinical data to maximize its utility.

#### Near-Infrared Spectroscopy

Unlike ultrasound, NIRS has the potential to directly penetrate bone and several centimeters into underlying tissue. Compared to ultrasound, NIRS is better suited to a small form factor and thus is potentially closer to becoming a reliable method for non-invasive cerebral monitoring in the prehospital setting. Previous reviews have covered the physics of NIRS and applications in TBI (58). Here, we will focus on the differences in continuous wave (CW) and frequency domain (FD) NIRS for detection of space occupying intracranial hematomas.

To detect intracranial hematomas, NIRS relies on the principle that light is readily absorbed by extravascular blood due to the concentration of hemoglobin. Of the NIRS detection schemes, the CW mode is the simplest to implement and measures the attenuation of light through tissue. Because no absolute measurements are provided, a reference value—typically from the contralateral side after a bilateral scan—is required to determine the presence of extravascular blood. Most diffuse optics research and technology, including the vast majority of functional brain imaging systems, has focused on CW NIRS measurements (59).

Designed to detect hematomas >3.5 cc in volume <2.5 cm from the brain surface, early studies with the CW-based Infrascanner™ demonstrated sensitivities of 88–90% and specificities of 81–91% (41, 42). Subsequent studies with newer models demonstrated similar or better accuracy (43–46), and a recent meta-analysis found a cross-study sensitivity of 92.5 and 93% and specificity of 82.9 and 86.5% in adults and children, respectively (60). However, a 2017 meta-analysis of 8 studies using the Infrascanner™ found a cross-study sensitivity of 78%, a specificity of 90%, a positive predictive value of 77%, and a negative predictive value of 90% (61). A recent far-forward military user evaluation also reported concerns about the operational efficacy and suitability of CW NIRS devices (62). Nevertheless, CW-based diagnostic devices have seen adoption among military units, ambulance services, and in hospitals as a bedside diagnostic tool.

A limitation of CW systems is that they can only be used to make a differential measurement (i.e., determine that something has changed over time or space), which limits their utility to localized injuries. Typically, bilateral measurements over the four lobes of the brain are made, and a difference in optical density between sides indicates the presence of hematoma. Although less common that unilateral hematomas, bilateral hematomas are an important subset of severe TBI and can be missed with CW-based diagnostics if the hematomas are both bilateral and symmetrical, due to equal absorption of light at each site. In an early study with a prototype CW NIRS device, bilateral hematomas were not detected in two patients because there was no change in optical density between readings (63). However, this represented a very small percentage of the 191 hematoma-positive patients in that study.

In contrast, FD systems allow for an absolute measurement of the optical properties of the sample under study by measuring information related to the time of flight of the photons through the sample. This is a major potential advantage over existing technologies. When tomographic reconstruction algorithms are coupled with FD measurements made using light sources and detectors at multiple positions across the sample, a 3D reconstruction of the target's optical properties can be generated to enable spatial localization of the pathology with centimeter resolution. This type of information derived from FD-diffuse optical tomography (FD-DOT) systems has shown great potential as a diagnostic tool for breast tumor imaging and other diagnostics (64, 65). These systems have demonstrated the ability of DOT to detect even small changes in oxy- and deoxyhemoglobin concentrations non-invasively at up to 2 cm depth from the surface of the head (59). Therefore, DOT appears to meet important criteria for solving the problem of accurately detecting both the presence and spatial location of intracranial hematoma. This capability can lead to more aggressive management at the point of injury, and spatial localization of intracranial hematoma may ultimately lead to the development of prehospital surgical intervention in the near future.

Thus, far, intracranial hematoma detection has not been an active area of investigation for FD NIRS systems, and thus any potential advantages remain theoretical. Furthermore, FD systems are not without limitations. Given the additional system complexity necessitated by FD compared to CW systems, current state-of-the-art FD systems are generally large and require external sources of power, which would limit their utility in austere, remote, or rural operational environments. However, recent results from development and testing of key FD systems components for portable systems have been presented, which could lead to a smaller form factor in the future (66, 67). Compared to CW systems, they are also more costly, potentially limiting their use in low- and middle-income countries where the number of severe TBIs is on the rise.

An unresolved problem for using NIRS to detect intracranial hematoma is navigating complex pathologies such as intracranial hematoma coexistent with subgaleal hematoma, soft tissue swelling, and scalp laceration. The ability to “see through” a superficial hematoma has not yet been solved, and thus superficial bleeding raises concerns for false positives. In certain operational environments, such as on the battlefield or in wilderness medicine situations involving inclement weather, false positives may trigger unnecessary medical evacuation, placing personnel at risk (68).

### Preclinical Models

The technologies for non-invasive cerebral monitoring and diagnosis discussed herein will require a substantial amount of additional investigation, including foundational work in animal models. Animal studies are frequently an important step on the path to translation of investigational devices. However, the inability to translate findings from animal studies to clinical trial methodology is frequently cited as a reason for the more than 30 late-phase failed clinical trials in TBI (69–71). While the majority of preclinical research has been conducted in rodents, the behavioral and physiological outcomes most relevant to severe TBI are best measured in large animals with gyrencephalic brains, including pigs and sheep. Large animal models of life-threatening severe TBI, such as the SDH models in pigs (72, 73), will be critical for assessing physiological parameters relevant to severe TBI. A number of translational studies in pigs have demonstrated the importance of physiological monitoring and functional assessment in predicting outcome following severe TBI (74–76). The sheep may be an especially promising model for developing ultrasound and NIRS-based technologies for non-invasive diagnosis, as the skull thickness, porosity, and curvature is closer to that of humans than other large animals, including pigs (77).

### Training Prehospital Providers

While material solutions and emerging technologies offer hope for enhanced diagnosis and monitoring of severe TBI in remote settings, it is critical that investments in technology are balanced with those applied to training of prehospital providers. Indeed, many of the life-saving advances in prehospital care over the previous decades have been made not in material development, but in improved training and trauma systems development. Simulation-based training in particular incorporates real-life scenarios with real-life time constraints and will be critical for evaluating new technologies designed for the field. Simulation training improves crisis management skills (78), and both technical and non-technical skills needed to manage severely injured patients can be reliably assessed by independent observers (79). Trauma simulation training has been shown to improve confidence in performing life-saving procedures in the prehospital setting (80).

Training prehospital providers to use novel medical technologies in the field must be balanced with the more traditional role of providing basic life support while ensuring swift transport to higher-level facilities when available. Current data do not universally support the notion that providing advanced care or diagnosis at the scene of the injury improves outcomes (81), although rural environments would presumably see greater benefits. For instance, in moderate to severe TBI, prehospital intubation has been associated with increased mortality in some studies (82, 83), and this has been attributed to the skill and experience level of the paramedics performing the intubation (84). Nevertheless, even fully-resourced settings, such as tertiary care centers, will benefit from more rapid diagnosis, triage, and management. The ability to deploy new technology efficiently will depend on intensive initial training and the maintenance of skillsets through repeat usage or recurrent training and simulation. Paradoxically, highly-trained prehospital providers frequently work in urban environments where the volume of cases allows frequent practice of skills; providers with only basic training are more likely to work in rural environments, where the stakes are often higher due to the geographic separation from specialized care (85). The relatively low volume of cases in rural areas will make the maintenance of skills a real issue.

Ultimately, deploying new medical devices in the prehospital setting must balance the perceived safety and efficacy of the new technology with a realistic appraisal of the social, legal, and organizational context in which they will be deployed (86). Resistance to implementing potentially life-saving technologies, including both idiosyncratic alterations of the intended use and deliberate non-use, has been observed following the introduction of new prehospital technologies (86). Thus, introduction of new technologies should be seen as an ongoing process with involvement from stakeholders at all levels. Knowing when not to deploy a new technology may be as important as knowing when they are useful. For instance, until appropriate therapeutics are available, the care of some severely injured patients *en route* to high level care facilities may not change based on the results of a diagnostic scan (43). Computational modeling can help determine the appropriate geographic parameters for implementing advanced care or diagnostics at the injury scene vs. engaging in swift transport to higher level facilities (87). Similarly, identifying the presence and size of a hematoma can better inform triage decisions. For example, one recent study reported that larger hematomas over 15 mm in thickness are associated with higher mortality (88). There may be some cases that are judged to be so severe that urgent evacuation or *in situ* aggressive management may be considered futile. Thus, implementation science and appropriate patient management skills may be just as important as technology development for improving prehospital care for severe TBI in austere, rural, and remote areas.

## Discussion

There has been little improvement in mortality associated with severe TBI in the past 30 years (89). Nearly 40% of patients with severe TBI die from their injury. Although robust data on preventable deaths is not widely available, a significant number of preventable deaths likely occur in the prehospital setting (90). Improvement in mortality rates and patient outcomes following severe TBI in rural and remote settings will require a multidisciplinary approach that involves developing new technologies and leveraging existing ones. There is a great need for simple-to-use, portable, and easy-to-maintain devices capable of detecting life-threatening hemorrhage and other intracranial pathologies. Although not discussed here, blood-based biomarkers have been an area of intense investigation in recent years and may be useful for detection of life-threatening post-injury complications including intracranial hypertension (91) or evolving intracranial lesions (92).

The choice to deploy point-of-care diagnostics in the prehospital setting will depend on a number of factors. First, the operational environment will dictate, to a certain extent, which diagnostics or technologies are feasible. While ultrasound-based technologies may be appropriate to implement in a rural ambulance service, they are less likely to be used by far-forward military personnel, who will favor handheld devices or technologies that can be readily integrated into existing medical kits. Second, the lack of empirical data from controlled studies remains a critical barrier to implementing potentially life-saving technologies in the prehospital setting. Successfully implementing these technologies in the prehospital setting will require extensive development and validation in the laboratory, assessment in clinically-relevant animal models, and rigorous and ongoing training for the ultimate end-users. End-user feedback from real-world operational environments will remain an indispensable component of technology adoption and should occur early and often (62). The sensitivity and specificity of each diagnostic should be evaluated rigorously in prospective studies. Randomized controlled trials will remain the gold standard for determining success or failure of new technologies, and a commitment to publishing results—both positive and negative—will lead to improved understanding of the fundamental pros and cons of each technology.

## Data Availability Statement

The original contributions presented in the study are included in the article/supplementary material, further inquiries can be directed to the corresponding author/s.

## Author Contributions

DLB conceptualized the paper. MW, BD, and DLB wrote the initial draft of the manuscript. All authors critically reviewed the manuscript for intellectual content and approved the final version of the manuscript for submission.

## Conflict of Interest

AJ was employed by Knowesis, Inc. The remaining authors declare that the research was conducted in the absence of any commercial or financial relationships that could be construed as a potential conflict of interest.
